# Long-Term Fine Particulate Matter Exposure and Major Depressive Disorder in a Community-Based Urban Cohort

**DOI:** 10.1289/EHP192

**Published:** 2016-04-29

**Authors:** Kyoung-Nam Kim, Youn-Hee Lim, Hyun Joo Bae, Myounghee Kim, Kweon Jung, Yun-Chul Hong

**Affiliations:** 1 Department of Preventive Medicine, Seoul National University College of Medicine, Seoul, Republic of Korea; 2 Institute of Environmental Medicine, Seoul National University Medical Research Center, Seoul, Republic of Korea; 3 Environmental Health Center, Seoul National University College of Medicine, Seoul, Republic of Korea; 4 Korea Environment Institute, Sejong, Republic of Korea; 5 Department of Dental Hygiene, College of Health Science, Eulji University, Gyeonggi-do, Republic of Korea; 6 Seoul Metropolitan Institute of Public Health and Environment, Seoul, Republic of Korea

## Abstract

**Background:**

Previous studies have associated short-term air pollution exposure with depression. Although an animal study showed an association between long-term exposure to particulate matter ≤ 2.5 μm (PM2.5) and depression, epidemiological studies assessing the long-term association are scarce.

**Objective:**

We aimed to determine the association between long-term PM2.5 exposure and major depressive disorder (MDD).

**Methods:**

A total of 27,270 participants 15–79 years of age who maintained an address within the same districts in Seoul, Republic of Korea, throughout the entire study period (between 2002 and 2010) and without a previous MDD diagnosis were analyzed. We used three district-specific exposure indices as measures of long-term PM2.5 exposure. Cox proportional hazards models adjusted for potential confounding factors and measured at district and individual levels were constructed. We further conducted stratified analyses according to underlying chronic diseases such as diabetes mellitus, cardiovascular disease, and chronic obstructive pulmonary disease.

**Results:**

The risk of MDD during the follow-up period (2008–2010) increased with an increase of 10 μg/m3 in PM2.5 in 2007 [hazard ratio (HR) = 1.44; 95% CI: 1.17, 1.78], PM2.5 between 2007 and 2010 (HR = 1.59; 95% CI: 1.02, 2.49), and 12-month moving average of PM2.5 until an event or censor (HR = 1.47; 95% CI: 1.14, 1.90). The association between long-term PM2.5 exposure and MDD was greater in participants with underlying chronic diseases than in participants without these diseases.

**Conclusion:**

Long-term PM2.5 exposure increased the risk of MDD among the general population. Individuals with underlying chronic diseases are more vulnerable to long-term PM2.5 exposure.

**Citation:**

Kim KN, Lim YH, Bae HJ, Kim M, Jung K, Hong YC. 2016. Long-term fine particulate matter exposure and major depressive disorder in a community-based urban cohort. Environ Health Perspect 124:1547–1553; http://dx.doi.org/10.1289/EHP192

## Introduction

Major depressive disorder is characterized by persistent low mood, loss of interest, fatigue, and low energy, lasting for at least 2 weeks, and is one of the most common mental illnesses and a major cause of disability worldwide (Moussavi et al. 2007). In the United States, the lifetime prevalence of major depressive disorder is estimated to be 18.6% and the 1-year prevalence 8.1% (González et al. 2010), and in the Republic of Korea, these are estimated to be 6.7% and 3.1%, respectively (Cho 2011).

Previous studies have identified an association between short-term air pollution exposure (for weeks or shorter) and depression. A time-series study reported an association between air pollution exposure and emergency department visits for depression, based on the discharge diagnosis from 11 hospitals in six cities in Canada (Szyszkowicz et al. 2009). A panel study performed among noninstitutionalized elderly individuals demonstrated an association between air pollution exposure and depressive symptoms, as evaluated with a questionnaire-based assessment tool (Lim et al. 2012). Additionally, a case-crossover study presented an association between air pollution exposure and emergency department visits for depression using medical claims data reported to the National Health Insurance program of the Republic of Korea (Cho et al. 2014). Moreover, studies have identified associations between short-term air pollution exposure and suicide completion using mortality data provided by the Department of Health’s Office of the Medical Examiner (Bakian et al. 2015) and data on suicide attempts using diagnosis information from one emergency department in Canada (Szyszkowicz et al. 2010).

Although there is accumulating evidence for the effects of long-term air pollution exposure (for months or longer) on neuropsychological function (reviewed by Tzivian et al. 2015), few studies have been conducted on the association between long-term air pollution exposure and depression. An experimental study reported an association between long-term exposure to fine particulate matter (particles with an aerodynamic diameter ≤ 2.5 μm; PM_2.5_) and depressive-like behaviors in mice (Fonken et al. 2011). However, to our knowledge, no epidemiological study has investigated the association between long-term air pollution exposure and depression, except for a study conducted among elderly individuals in Boston, Massachusetts, that assessed depressive symptoms using a questionnaire-based instrument, which did not identify any association (Wang et al. 2014).

Among various air pollutants, PM_2.5_ has been demonstrated to be especially relevant for neuropsychological outcomes because it is capable of reaching the brain owing to its small size (Block and Calderón-Garcidueñas 2009; Calderón-Garcidueñas et al. 2015). PM_2.5_ has been suggested to affect the central nervous system directly or indirectly through inflammatory processes. Because systemic and neural inflammation play important roles in the development of major depressive disorder (Anisman and Hayley 2012), PM_2.5_ exposure—particularly long-term exposure—may increase the risk of major depressive disorder. Furthermore, underlying diseases related to chronic inflammation such as diabetes mellitus, cardiovascular disease, and chronic obstructive pulmonary disease may enhance this association, which has been suggested in previous epidemiological studies (Cho et al. 2014; Kim et al. 2010).

Although there have been a few studies linking air pollution exposure with emergency department visits for depression or depressive symptoms evaluated by a questionnaire, to our knowledge, major depressive disorder has not been assessed as outcome yet. Furthermore, most previous studies have focused on short-term air pollution exposure. Therefore, in the present study, we investigated the association between long-term PM_2.5_ exposure and major depressive disorder among the general population in Seoul, Republic of Korea. We also evaluated the effect modification of the association between PM_2.5_ exposure and major depressive disorder by underlying chronic diseases such as diabetes mellitus, cardiovascular disease, and chronic obstructive pulmonary disease.

## Methods

### Study Population and Design

The present study was performed between 1 January 2002 and 31 December 2010, using data from the National Health Insurance database (NHID) (*n* = 1,025,340 in 2002), which includes a proportionate stratified random sample of individuals who visited hospitals under the Korea National Health Insurance program that covers all legal residents of the Republic of Korea. The NHID contains demographic information, including age, sex, household income, and district-level address, and inpatient and outpatient medical care utilization information, including date of service, disease diagnosed (*International Classification of Diseases, 10th Revision*; ICD-10), drugs prescribed, and medical or surgical procedures performed. The database also includes the health examination results of individuals who participated in free health examination services provided by the National Health Insurance. Individuals with lower income and disability have been reported to be less likely to participate in the free health examination than those with higher income and without disability (Cho and Lee 2011; Park et al. 2006).

Of 85,758 individuals from the NHID who maintained an address within the same districts in Seoul during the study period, only those who underwent health examinations between 1 January 2002 and 31 December 2007, 15–79 years of age, and without major depressive disorder between 1 January 2002 and 31 December 2007 were included. Thus, a total of 27,270 individuals were finally enrolled (see Figure S1).

The identification numbers of the participants were encrypted before data use, and district-level addresses were used to protect private information. The Institutional Review Board of Seoul National University Hospital reviewed and approved the study protocol (IRB no. E-1407-001-588).

### Definition of Major Depressive Disorder

**Figure 1:**
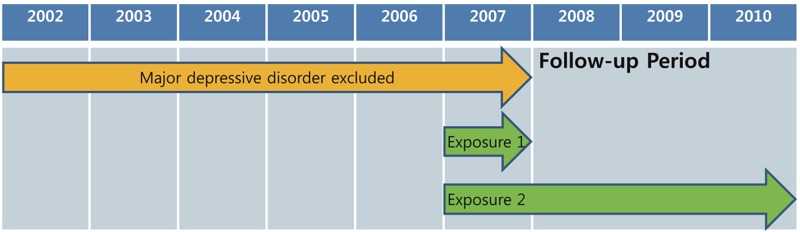
Schematic representation of the study period. Exposure 1: average PM_2.5_ concentration in 2007; exposure 2: average PM_2.5_ concentration between 2007 and 2010. PM_2.5_, particulate matter with an aerodynamic diameter ≤ 2.5 μm.

The first recorded diagnosis of major depressive disorder, defined as a diagnosis of depressive episodes (ICD-10 code F32.x) along with antidepressant prescription (selective serotonin reuptake inhibitors, tricyclic antidepressants, monoamine oxidase inhibitors, serotonin noradrenaline reuptake inhibitors, or other antidepressants) during the follow-up period, was used as the primary outcome in the present study (Figure 1). In the sensitivity analyses, the outcome was differently defined according to the following ICD-10 codes, without antidepressant prescription: *a*) F32.x or *b*) F32.x, F33.x (recurrent depressive disorder), F34.1 (dysthymia), or F41.2 (mixed anxiety and depressive disorder).

### Assessment of PM_2.5_ Exposure

Seoul is the capital and largest city in the Republic of Korea, and it includes 25 districts with areas ranging from 9.96 to 47.00 km^2^ (mean, 24.21 km^2^). Since 2007, PM_2.5_ monitoring data have been available for the 25 districts in Seoul from 27 monitoring sites (2 districts had 2 monitoring sites). The PM_2.5_ monitoring data were obtained from the Research Institute of Public Health and Environment in Seoul. The following district-specific exposure indices were used as measures of long-term PM_2.5_ exposure: *a*) the average PM_2.5_ concentration in 2007 (1 year before the follow-up period), *b*) average PM_2.5_ concentration between 2007 and 2010 (1 year before the follow-up period and 3 years of the follow-up period), and *c*) 12-month moving average of the PM_2.5_ concentration until an event or censor between 2007 and 2010 as time-varying exposure (Figure 1). These exposure indices were calculated using the daily average estimated from the 24-hr monitoring data obtained from the 27 monitoring sites. In the districts with 2 monitoring sites, the PM_2.5_ concentrations measured at the 2 sites were averaged. The PM_2.5_ measurements were based on the gravimetric method and followed the standard reference protocols established by the Korean Ministry of Environment (MOE 2011).

### Statistical Analysis

Cox proportional hazards models were constructed with sex and age (5-year groups; 15–79 years) as the stratification variables, with each stratum having its own baseline hazard. The follow-up time was calculated in months from 1 January 2008, to the date of death, case occurrence, or follow-up end (31 December 2010), whichever occurred first. The average PM_2.5_ concentration in 2007 and PM_2.5_ concentration between 2007 and 2010 were used as measures of PM_2.5_ exposure. Additionally, we considered the 12-month moving average of the PM_2.5_ concentration until an event or censor between 2007 and 2010 as time-varying exposure in time-dependent Cox models. We also conducted nonparametric analyses for estimating hazard ratio curves using spline smoothing methods to evaluate the shape of the association between PM_2.5_ concentration and major depressive disorder (Meira-Machado et al. 2013).

We specified the models adjusted for potential confounders selected *a priori*. Cox models stratified by sex and age were adjusted for individual-level covariates: household income (categorized into deciles), smoking status (nonsmoker, ex-smoker, smoker, or did not answer), alcohol consumption (no alcohol consumption, < 1/week, 1 or 2/week, 3–4 times/week, > 4 times/week, or did not answer), and regular exercise (no exercise, 1 or 2/week, 3–4 times/week, 5–6 times/week, almost every day, or did not answer). Individual-level information, except household income, was obtained from the health examination results. The models were also adjusted for contextual covariates: the size of the population, proportion of married persons among the population ≥ 15 years of age, economic environment satisfaction (10-point Likert scale), social environment satisfaction (10-point Likert scale), number of clients of the social welfare facilities per capita, and deprivation index (Shin et al. 2009) in the district in which each participant resided. Contextual information was obtained from the Korean Statistical Information Service. For participants with multiple health examination results, the results closest to the follow-up period (2008–2010) were used to identify the individual-level covariates. We used the results of health examinations conducted between 2002 and 2007. Most (73.2%) of the results used as individual-level covariates were from health examinations performed in 2006 and 2007. Information of all contextual covariates were obtained in 2007, except the proportion of married persons among the population ≥ 15 years of age, which was obtained in 2005, and the deprivation index, which was obtained in 2010, owing to availability of data. Covariates with missing values were included in the model with a missing indicator category.

Stratified analyses were performed according to the following underlying chronic diseases diagnosed between 1 January 2002 and 31 December 2007: diabetes mellitus (ICD-10 codes E10.x–E14.x), cardiovascular disease including hypertension (I10.x–I15.x, I20.x–I25.x, or I60.x–I69.x), and chronic obstructive pulmonary disease (J40–J44.x). Stratified analyses were also conducted by household income level, categorized as 0–3 (low), 4–6 (middle), and 7–10 (high) deciles of household income.

Several sensitivity analyses were performed. First, additional contextual covariates (birth rate, the ratio of paved roadway to the area of the specific urban district, and the number of beneficiaries of public assistance per capita) were further adjusted to control for potential district-related confounding. Second, multilevel time-dependent Cox models with a random effect of districts and a time-dependent Cox model further adjusted for the district in which each participant resided were constructed. These analyses using intra-district variability of PM_2.5_ concentration as exposure contrast were conducted to consider potential clustering and confounding by districts. Third, different outcome definitions were used to confirm that the results were robust with the outcome definitions and were not driven by the specific set of disease codes. Fourth, participants diagnosed with dementia (ICD-10 codes G30.x, G31.x, and F00.x–F03) during the study period were excluded to avoid misclassification bias and potential confounding. Fifth, the association was reanalyzed after including individuals who had major depressive disorder between 1 January 2002 and 31 December 2007. Sixth, participants who died from intentional self-injury (ICD-10 codes X60–X84) during the follow-up period were considered to have major depressive disorder and were included in the analysis. Seventh, the association in the population with and without health examination results was assessed to evaluate the generalizability of the findings to larger populations. Eighth, we conducted complete case analyses due to the potential bias by using the missing indicator category.

All analyses were conducted using SAS version 9.4 (SAS Institute Inc.) and R version 3.1.0 (R Core Team 2015). In all analyses, a *p*-value < 0.05 was considered significant.

## Results

Among 85,758 individuals who maintained an address within the same districts in Seoul during the study period, 27,270 were included in the present analyses. Males and participants 40–69 years of age were more likely to be included in the analyses compared to those who were excluded (see Table S1). The demographic characteristics of the 27,270 participants are presented in Table 1. Of these participants, 54% were male, and the majority of participants were 40–59 years of age. A high number of participants came from families with high income, and did not smoke tobacco, drink alcohol, or exercise regularly.

**Table 1:** Descriptive characteristics of the study participants (*n* = 27,270) and the annual PM_2.5_ concentration in 2007.

Variable	Total (*n* = 27,270)^*a*^	PM_2.5_ (mean ± SD)	Individuals with MDD (*n* = 973)^*a*^	PM_2.5_ (mean ± SD)	Individuals without MDD (*n* = 26,297)^*a*^	PM_2.5_ (mean ± SD)
Sex
Male	14,782 (54)	29.9 ± 3.5	369 (38)	30.3 ± 3.4	14,413 (55)	29.9 ± 3.5
Female	12,488 (46)	29.8 ± 3.5	604 (62)	30.2 ± 3.5	11,884 (45)	29.8 ± 3.5
Age (years)
15–29	2,213 (8)	29.8 ± 3.5	32 (3)	29.5 ± 4.1	2,181 (8)	29.8 ± 3.5
30–39	4,288 (16)	29.7 ± 3.6	62 (6)	30.0 ± 3.6	4,226 (16)	29.7 ± 3.6
40–49	8,059 (30)	30.0 ± 3.5	247 (25)	30.5 ± 3.3	7,812 (30)	30.0 ± 3.5
50–59	7,445 (27)	29.9 ± 3.5	298 (31)	30.4 ± 3.4	7,147 (27)	29.9 ± 3.5
60–69	4,050 (15)	29.7 ± 3.5	222 (23)	30.0 ± 3.5	3,828 (15)	29.6 ± 3.5
70–79	1,215 (4)	29.4 ± 3.6	112 (12)	30.2 ± 3.3	1,103 (4)	29.4 ± 3.6
Household income (decile)
0–2	3,530 (13)	29.8 ± 3.6	131 (13)	30.6 ± 3.0	3,399 (13)	29.8 ± 3.6
3–4	3,708 (14)	29.8 ± 3.6	135 (14)	30.3 ± 3.4	3,573 (14)	29.8 ± 3.6
5–6	4,538 (17)	29.8 ± 3.5	136 (14)	29.8 ± 3.7	4,402 (17)	29.8 ± 3.5
7–8	5,724 (21)	29.8 ± 3.6	198 (20)	30.2 ± 3.6	5,526 (21)	29.8 ± 3.6
9–10	9,770 (36)	29.9 ± 3.5	373 (38)	30.2 ± 3.4	9,297 (36)	29.9 ± 3.5
Smoking status
Nonsmoker	17,820 (65)	29.8 ± 3.5	742 (76)	30.2 ± 3.5	17,078 (65)	29.8 ± 3.5
Ex-smoker	1,588 (6)	30.0 ± 3.6	39 (4)	30.0 ± 3.3	1,549 (6)	30.0 ± 3.6
Smoker	6,442 (24)	29.9 ± 3.5	144 (15)	30.4 ± 3.4	6,298 (24)	29.9 ± 3.5
Did not answer	1,420 (5)	29.9 ± 3.4	48 (5)	30.4 ± 3.1	1,372 (5)	29.9 ± 3.4
Alcohol consumption
No alcohol consumption	13,627 (50)	29.8 ± 3.5	627 (64)	30.2 ± 3.4	13,000 (49)	29.8 ± 3.5
Less than once/week	5,280 (19)	30.0 ± 3.5	139 (14)	30.8 ± 3.5	5,141 (20)	29.9 ± 3.5
Once or twice/week	5,390 (20)	29.9 ± 3.6	130 (13)	30.1 ± 3.7	5,260 (20)	29.9 ± 3.6
3–4 times/week	1,939 (7)	29.8 ± 3.5	46 (5)	30.2 ± 2.7	1,893 (7)	29.8 ± 3.5
More than 4 times/week	793 (3)	29.6 ± 3.4	20 (2)	28.8 ± 4.3	773 (3)	29.6 ± 3.4
Did not answer	241 (1)	30.0 ± 3.5	11 (1)	31.3 ± 2.4	230 (1)	30.0 ± 3.6
Regular exercise
No exercise	13,354 (49)	29.8 ± 3.6	476 (49)	30.1 ± 3.5	12,878 (49)	29.7 ± 3.6
Once or twice/week	7,956 (29)	29.9 ± 3.5	249 (26)	30.4 ± 3.4	7,707 (29)	29.9 ± 3.5
3–4 times/week	3,534 (13)	30.0 ± 3.4	138 (14)	30.3 ± 3.2	3,396 (13)	30.0 ± 3.4
5–6 times/week	814 (3)	30.0 ± 3.5	37 (4)	30.7 ± 3.1	777 (3)	29.9 ± 3.5
Almost every day	1,329 (5)	29.8 ± 3.5	66 (7)	30.2 ± 3.5	1,263 (5)	29.7 ± 3.5
Did not answer	283 (1)	29.8 ± 3.4	7 (1)	29.6 ± 3.3	276 (1)	29.8 ± 3.5
Size of the population in the district^*b*^	445,095 (164,399)		445,095 (164,941)		445,095 (164,399)
Proportion of married persons among the population aged ≥ 15 years^*b*^	0.56 (0.04)		0.56 (0.04)		0.56 (0.04)
Economic environment satisfaction^*b,c*^	4.85 (0.45)		4.85 (0.46)		4.85 (0.45)
Social environment satisfaction^*b,c*^	5.08 (0.52)		5.08 (0.56)		5.08 (0.52)
Number of clients of social welfare facilities^*b,d*^	8.85 (4.22)		8.85 (5.64)		8.85 (4.22)
Deprivation index^*b*^	0.03 (2.53)		0.03 (2.52)		0.03 (2.53)
Abbreviations: MDD, major depressive disorder; PM_2.5_, particulate matter with an aerodynamic diameter ≤ 2.5 μm; SD, standard deviation. ^***a***^Values are presented as *n* (%) or median (interquartile range). ^***b***^Interquartile ranges, defined as third quartile minus first quartile. ^***c***^Evaluated as a 10-point Likert scale. ^***d***^Presented per 10,000 people.						

The annual average PM_2.5_ concentration in Seoul declined from 29.8 μg/m^3^ in 2007 to 24.9 μg/m^3^ in 2010, and the average PM_2.5_ concentration between 2007 and 2010 was 26.7 μg/m^3^. The variation in the annual PM_2.5_ concentrations among the districts also decreased during this period. In 2007, the average PM_2.5_ concentrations among the districts ranged from 20.3 μg/m^3^ to 34.5 μg/m^3^, and in 2010, the concentrations ranged from 19.8 μg/m^3^ to 27.4 μg/m^3^ (see Table S2 and Figure S2).

In Cox proportional hazards models with the baseline hazard function stratified by sex and age, PM_2.5_ exposure indices were associated with an increased risk of major depressive disorder after adjusting for contextual and individual-level covariates. The risk increased with an increase of 10 μg/m^3^ in the PM_2.5_ concentration in 2007 [hazard ratio (HR) = 1.44; 95% confidence interval (CI): 1.17, 1.78], PM_2.5_ concentration between 2007 and 2010 (HR = 1.59; 95% CI: 1.02, 2.49), and 12-month moving average of the PM_2.5_ concentration until an event or censor between 2007 and 2010 (HR = 1.47; 95% CI: 1.14, 1.90; Table 2). In the spline regression analysis, we found a linear positive association between PM_2.5_ concentration in 2007 and major depression disorder approximately above the level of annual average PM_2.5_ concentration in Seoul between 2007 and 2010 (26.7 μg/m^3^; see Figure S3). The association was greater among middle-aged and elderly participants than among the other participants (see Table S3).

**Table 2:** Hazard ratios of major depressive disorder for an increase of 10 μg/m^3^ in the PM_2.5_ concentration.

Exposure	No.^*a*^	HR (95% CI)
Model 1^*b*^
PM_2.5_ in 2007	973/27,270	1.50 (1.24, 1.81)
PM_2.5_ between 2007 and 2010		1.61 (1.06, 2.44)
Moving average PM_2.5_^*c*^		1.43 (1.12, 1.84)
Model 2^*d*^
PM_2.5_ in 2007	973/27,270	1.50 (1.24, 1.81)
PM_2.5_ between 2007 and 2010		1.60 (1.06, 2.43)
Moving average PM_2.5_^*c*^		1.44 (1.12, 1.84)
Model 3^*e*^
PM_2.5_ in 2007	973/27,270	1.43 (1.16, 1.77)
PM_2.5_ between 2007 and 2010		1.59 (1.02, 2.49)
Moving average PM_2.5_^*c*^		1.47 (1.13, 1.89)
Model 4^*f*^
PM_2.5_ in 2007	973/27,270	1.44 (1.17, 1.78)
PM_2.5_ between 2007 and 2010		1.59 (1.02, 2.49)
Moving average PM_2.5_^*c*^		1.47 (1.14, 1.90)
Abbreviations: CI, confidence interval; HR, hazard ratio; PM_2.5_, particulate matter with an aerodynamic diameter ≤ 2.5 μm. ^***a***^Number of events during the follow-up period/total number analyzed. ^***b***^Model 1 was stratified by sex and age, and unadjusted. ^***c***^The 12-month moving average of the PM_2.5_ concentration until an event or censor between 2007 and 2010 as time-varying exposure in time-dependent Cox models. ^***d***^Model 2 was stratified by sex and age, and adjusted for household income, smoking status, alcohol consumption, and regular exercise. ^***e***^Model 3 was stratified by sex and age, and adjusted for the size of the population, proportion of married persons among the population ≥ 15 years of age, economic and social environment satisfaction, number of clients of the social welfare facilities per capita, and deprivation index in the district in which each participant resided. ^***f***^Model 4 was stratified by sex and age, and adjusted for household income, smoking status, alcohol consumption, regular exercise, size of the population, proportion of married persons among the population ≥ 15 years of age, economic and social environment satisfaction, number of clients of the social welfare facilities per capita, and deprivation index in the district in which each participant resided.		

When stratified by underlying chronic disease, the association between PM_2.5_ exposure and major depressive disorder was greater in participants with diabetes mellitus (HR = 1.83; 95% CI: 1.26, 2.64) than in those without (HR = 1.27; 95% CI: 0.98, 1.64; *p*-value for interaction = 0.12). Similar associations were identified for cardiovascular disease and chronic obstructive pulmonary disease (Table 3). When we stratified the participants according to household income level, the association was stronger among those with low income (0–3 deciles) than among those with higher income (≥ 4 deciles) (see Table S4).

**Table 3:** Hazard ratios*^a^* of major depressive disorder for an increase of 10 μg/m^3^ in the annual PM_2.5_ concentration in 2007, stratified by underlying chronic diseases.

Disease	No.^*b*^	HR (95% CI)	*p* for interaction
DM
(+)	350/5,252	1.83 (1.26, 2.64)	0.12
(–)	623/22,018	1.27 (0.98, 1.64)
CVD
(+)	506/8,564	1.58 (1.19, 2.12)	0.64
(–)	467/18,706	1.31 (0.96, 1.77)
COPD
(+)	389/7,530	1.64 (1.17, 2.30)	0.44
(–)	583/19,740	1.35 (1.03, 1.77)
Abbreviations: +, individuals with the underlying disease; –, individuals without the underlying disease. CI, confidence interval; COPD, chronic obstructive pulmonary disease; CVD, cardiovascular disease; DM, diabetes mellitus; HR, hazard ratio; PM_2.5_, particulate matter with an aerodynamic diameter ≤ 2.5 μm. ^***a***^The models were stratified by sex and age, and adjusted for household income, smoking status, alcohol consumption, regular exercise, size of the population, proportion of married persons among the population ≥ 15 years of age, economic and social environment satisfaction, number of clients of the social welfare facilities per capita, and deprivation index in the district in which each participant resided. ^***b***^Number of events during the follow-up period/total number analyzed, stratified by underlying chronic diseases.			

Several sensitivity analyses were performed. First, additional adjustment for contextual covariates did not change the results appreciably (HR = 1.45; 95% CI: 1.14, 1.84 for the PM_2.5_ concentration in 2007; HR = 1.42; 95% CI: 0.87, 2.31 for the PM_2.5_ concentration between 2007 and 2010; HR = 1.40; 95% CI: 1.07, 1.84 for the 12-month moving average of the PM_2.5_ concentration until an event or censor between 2007 and 2010). Second, in multilevel models with a random effect of districts, the results were robust (see Table S5). When districts were further adjusted in the time-dependent Cox model, the association remained consistent (HR = 1.42; 95% CI: 1.02, 1.98). Third, the results were robust when different outcome definitions were used (see Table S6). In these analyses, the denominator varied, because the number of individuals with depressive disorder between 1 January 2002 and 31 December 2007 differed according to the outcome definitions. Fourth, after excluding participants diagnosed with dementia, the results did not change appreciably (HR = 1.39; 95% CI: 1.12, 1.74 for the PM_2.5_ concentration in 2007; HR = 1.56; 95% CI: 0.98, 2.48 for the PM_2.5_ concentration between 2007 and 2010; HR = 1.39; 95% CI: 1.06, 1.81 for the 12-month moving average of the PM_2.5_ concentration until an event or censor between 2007 and 2010). Fifth, the association was consistent after including individuals who had major depressive disorder between 2002 and 2007 (see Table S7). Sixth, after including individuals who died from intentional self-injury, the results did not change appreciably (see Table S8). Seventh, the results were robust among the population with and without health examination results, after adjusting for household income and contextual covariates (see Table S9). Eighth, the results did not change appreciably in the complete case analyses (data not shown).

## Discussion

In the present community-based urban cohort study, we found an association between long-term PM_2.5_ exposure and major depressive disorder. Additionally, the risk for major depressive disorder was higher in participants with underlying chronic diseases than in those without these diseases.

To our knowledge, there has been only one epidemiological study investigating the association between long-term air pollution exposure and depression to date (Wang et al. 2014). This study, performed among elderly individuals in Boston, Massachusetts, found no evidence of an association between long-term air pollution exposure and depressive symptoms. Although this study and the present study considered PM_2.5_ as the exposure of interest, particulate composition, which has been shown to cause heterogeneity in health effects (Kioumourtzoglou et al. 2015), may differ between the studies, resulting in inconsistent results. Different PM_2.5_ concentrations (8.6 μg/m^3^ in the previous study vs. 26.7 μg/m^3^ in the present study) may also be responsible for the inconsistent results, with our penalized regression spline model showing that there was a PM_2.5_ concentration at which an association was not observed (see Figure S3). Additionally, differences in demographic characteristics such as age and race, underlying disease status, main outcome, and study design may induce heterogeneity and make a direct comparison difficult. Although the present study provides evidence for the association between long-term PM_2.5_ exposure and depression, there is still a lack of information regarding the potential different effects by particulate composition and nonlinearity of the association, which needs to be investigated in a future study.

The present study found that the association between long-term PM_2.5_ exposure and major depressive disorder was greater in participants with underlying chronic diseases than in those without these diseases. Previous studies that investigated the short-term effect of air pollution exposure have reported that underlying disease status may modify the association between air pollution exposure and depression or suicide. A case-crossover study reported an association between air pollution exposure and emergency department visits for depressive episodes among participants with chronic diseases such as diabetes mellitus, cardiovascular disease, chronic obstructive pulmonary disease, asthma, and depressive disorder, and found that this association was reduced in participants without these diseases (Cho et al. 2014). Another case-crossover study reported an association between air pollution exposure and suicide risk in participants with cardiovascular disease, and found no association among participants without cardiovascular disease (Kim et al. 2010). Our results are in line with the results of these reports, and imply that more attention should be paid to the effects of long-term PM_2.5_ exposure on depression among patients with underlying diseases such as diabetes mellitus.

Long-term PM_2.5_ exposure has been shown to induce inflammatory processes in the respiratory tract, resulting in systemic inflammation and circulation of inflammatory mediators (Calderón-Garcidueñas et al. 2009). These mediators may interact with cytokine receptors in brain endothelial cells, activate them, and generate autoantibodies to cell junction and neural proteins via immune system responses (Calderón-Garcidueñas et al. 2008; Lampron et al. 2013). These autoantibodies may disrupt the blood–brain barrier, leading to neuroinflammation, brain oxidative stress, and neurochemical changes. Additionally, it has been suggested that PM_2.5_ can penetrate the lung tissue compartment, enter systemic circulation, and reach the brain, leading to inflammation and oxidative stress in the central nervous system (Block and Calderón-Garcidueñas 2009; Valavanidis et al. 2008). There is evidence that inflammatory factors play important roles in the development of major depressive disorder through neurochemical changes (Anisman and Hayley 2012; Raison and Miller 2011). Major depressive disorder has been shown to frequently accompany diseases associated with chronic inflammation such as diabetes mellitus, cardiovascular disease, and chronic obstructive pulmonary disease (Shelton and Miller 2011). Additionally, the levels of circulating inflammatory mediators have been shown to be high in patients with major depressive disorder (Dantzer et al. 2008). Immunotherapy involving the administration of interferon-α for the treatment of hepatitis C or cancer has been shown to induce the production of proinflammatory cytokines such as interleukin-6 and depression among a considerable number of patients (Bonaccorso et al. 2002; Juengling et al. 2000). Therefore, it is biologically plausible that long-term PM_2.5_ exposure can cause major depressive disorder through systemic and neural inflammation and oxidative stress, and that the effect of PM_2.5_ exposure on major depressive disorder is great among patients with diseases associated with chronic inflammation because of the high levels of inflammatory factors. This effect modification may also reflect individual differences in susceptibility to inflammation caused by PM_2.5_ exposure. The development of chronic diseases related to chronic inflammation, such as diabetes mellitus and major depressive disorder, may be more likely in participants with a low defensive capacity against PM_2.5_ than in those with a normal defensive capacity (Rückerl et al. 2014). Further studies on the mechanisms should be performed.

Our study has limitations with regard to the study population. The present study was performed using data from a single metropolis with a population of > 10 million over an area of 605 km^2^, which may limit the generalizability of the findings. However, the racial and cultural homogeneity of the present study population reduced the possibility of residual confounding related to population stratification and cultural differences, which could not be excluded in previous studies assessing the adverse health effects of long-term air pollution exposure performed nationwide or in multiple cities. In the present study, a high number of participants came from families with high income, and those with low household income were underrepresented, which may shift the association to the null, considering that the association between PM_2.5_ exposure and major depressive disorder was stronger among those with low income in the stratified analyses by household income (see Table S4). There were missing values in up to 5.3% of individual-level covariates, which is another limitation of the present study. However, the results did not change appreciably in the analyses using the missing indicator category and in the complete case analyses.

Limitations regarding the assessment of exposure were present. Although assessing exposure using a fixed-site pollution monitor is common in air pollution epidemiological studies (Gauderman et al. 2015; Pope et al. 2009), this approach has been reported to cause nondifferential misclassification of individual exposure and to shift the association to the null (Kioumourtzoglou et al. 2014). However, despite this limitation, we found consistent associations in the present study. The possibility that unknown or unmeasured factors related to a district may confound the observed association cannot be excluded despite the relatively high racial and cultural homogeneity of the study population. Previous studies have demonstrated that income levels are closely related to the residential address and air pollution exposure (Brook et al. 2013; Koton et al. 2013), which is unlikely in the present study (Table 1). We tried to reduce this possibility by applying extensive contextual and individual-level covariates. Furthermore, when we constructed multilevel time-dependent Cox models with a random effect of districts or a time-dependent Cox model additionally adjusted for district, which assessed the association only with the within-district variability of exposure (Kioumourtzoglou et al. 2015; Park et al. 2015), the association was consistent, suggesting that the present results were not influenced notably by the potential confounders related to a district. Another possible source of misclassification of exposure in long-term air pollution studies is the methodology of assigning estimated exposure based on the home address of each participant at enrollment. Individuals who moved into their homes from other areas just before study enrollment or moved out from their homes to other areas during the study period may have a considerably different exposure to air pollution compared to the assigned exposure. Therefore, to reduce this kind of exposure misclassification, we restricted the analyses to participants who did not move to different districts between 2002 and 2010, because we obtained only the district-level address for each participant in each year in order to maintain confidentiality. Although the PM_2.5_ concentration was available only since 2007, we assumed that the structures related to pollution levels such as roads and factories changed gradually and that the ranking of the districts according to air pollution levels was relatively constant over the years (see Figure S2) (Dantzer et al. 2008; Park et al. 2015). Therefore, we used the annual PM_2.5_ concentration in 2007, average PM_2.5_ concentration between 2007 and 2010, and 12-month moving average of the PM_2.5_ concentration until an event or censor as the long-term exposure indices, reflecting > 1–4 years of exposure. However, in the present study, which used claims data, information on time spent away from the district in which each participant resided due to a reason such as work was not available, which may have resulted in misclassification of the estimation of individual PM_2.5_ exposure. Furthermore, PM_2.5_-related factors such as perceived air pollution/odor, perceived danger from road traffic, or traffic noise may also affect mental well-being (Bocquier et al. 2014; Persson et al. 2007; Yoshida et al. 1997) rather than, or together with, PM_2.5_ exposure, resulting in increased risk of major depressive disorder. These possibilities need to be investigated in a future study.

There were also limitations with regard to outcome assessment. The diagnostic accuracy of major depressive disorder in the present administrative data has not been evaluated. Therefore, to reduce the possibility of misclassification, we defined depressive disorder in the sensitivity analyses as the diagnosis of a depressive episode only or an extended diagnosis further including recurrent depressive disorder, dysthymia, and mixed anxiety and depressive disorder. The results were not appreciably different between the main definition and the alternative definitions, and showed gradual attenuation, possibly due to nondifferential misclassification (see Table S6). Long-term PM_2.5_ exposure has been reported to be associated with cognitive decline (Weuve et al. 2012) and neurodegenerative diseases such as Alzheimer’s disease (Jung et al. 2015) in the elderly, which can be a potential source of bias owing to misclassification. However, after excluding participants diagnosed with dementia, the results did not change appreciably. Last, the reported prevalence of major depressive disorder in Korea (1-year prevalence, 3.1%; lifetime prevalence, 6.7%) (Cho 2011) is comparable with our results (3-year prevalence, 5.1%; see Table S7), which provides additional evidence that the present outcome definition did not introduce substantial misclassification bias. Despite the inherent limitations of using claims data, this approach is more objective and potentially less biased than reporting by participants or a questionnaire-based instrument, which have been used in previous studies (MacIntyre et al. 2011).

The present study has some strengths. First, the study evaluated medical service utilization for major depressive disorder and treatment, which reflects clinical practice and real-world settings. Second, the study used claims data; therefore we avoided a systemic error due to differential follow-up loss, which is a potential problem when examining diseases whose development is related to follow-up and subsequent detection, such as major depressive disorder. Third, because both inpatient and outpatient medical care utilization information was used, inpatient participants with several medical conditions including major depressive disorder could be assessed, which is difficult in a usual cohort study setting.

## Conclusion

The present study provides evidence for a positive association between long-term PM_2.5_ exposure and major depressive disorder in a community-based urban cohort. The risk for major depressive disorder is higher in individuals with underlying chronic diseases who are exposed to high PM_2.5_ concentrations than in those without these diseases. However, because the present PM_2.5_ level was higher than the World Health Organization guideline for the annual mean PM_2.5_ concentration (10 μg/m^3^) and the PM_2.5_ levels of many developed countries, and because the shape of the concentration–response relationships may differ between areas due to heterogeneous populations and particulate composition, we could not generalize the present results directly to other areas. This provokes the necessity of further investigations confirming the observed associations among various population and areas.

## Supplemental Material

(442 KB) PDF

## References

[r1] Anisman H Hayley S 2012 Inflammatory factors contribute to depression and its comorbid conditions. Sci Signal 5 pe45, doi:10.1126/scisignal.2003579 23033537

[r2] Bakian AV Huber RS Coon H Gray D Wilson P McMahon WM 2015 Acute air pollution exposure and risk of suicide completion. Am J Epidemiol 181 295 303, doi:10.1093/aje/kwu341 25673816 PMC4339389

[r3] Block ML Calderón-Garcidueñas L 2009 Air pollution: mechanisms of neuroinflammation and CNS disease. Trends Neurosci 32 506 516, doi:10.1016/j.tins.2009.05.009 19716187 PMC2743793

[r4] Bocquier A Cortaredona S Boutin C David A Bigot A Sciortino V 2014 Is exposure to night-time traffic noise a risk factor for purchase of anxiolytic-hypnotic medication? A cohort study. Eur J Public Health 24 298 303, doi:10.1093/eurpub/ckt117 23985724

[r5] Bonaccorso S, Marino V, Biondi M, Grimaldi F, Ippoliti F, Maes M (2002). Depression induced by treatment with interferon-alpha in patients affected by hepatitis C virus.. J Affect Disord.

[r6] Brook RD Cakmak S Turner MC Brook JR Crouse DL Peters PA 2013 Long-term fine particulate matter exposure and mortality from diabetes in Canada. Diabetes Care 36 3313 3320, doi:10.2337/dc12-2189 23780947 PMC3781571

[r7] Calderón-Garcidueñas L Calderón-Garcidueñas A Torres-Jardón R Avila-Ramírez J Kulesza RJ Angiulli AD 2015 Air pollution and your brain: what do you need to know right now. Prim Health Care Res Dev 16 329 345, doi:10.1017/S146342361400036X 25256239

[r8] Calderón-Garcidueñas L Macías-Parra M Hoffmann HJ Valencia-Salazar G Henríquez-Roldán C Osnaya N 2009 Immunotoxicity and environment: immunodysregulation and systemic inflammation in children. Toxicol Pathol 37 161 169, doi:10.1177/0192623308329340 19171930

[r9] Calderón-Garcidueñas L Solt AC Henríquez-Roldán C Torres-Jardón R Nuse B Herritt L 2008 Long-term air pollution exposure is associated with neuroinflammation, an altered innate immune response, disruption of the blood-brain barrier, ultrafine particulate deposition, and accumulation of amyloid β-42 and α-synuclein in children and young adults. Toxicol Pathol 36 289 310, doi:10.1177/0192623307313011 18349428

[r10] Cho B, Lee CM (2011). Current situation of national health screening systems in Korea.. J Korean Med Assoc.

[r11] Cho J Choi YJ Suh M Sohn J Kim H Cho SK 2014 Air pollution as a risk factor for depressive episode in patients with cardiovascular disease, diabetes mellitus, or asthma. J Affect Disord 157 45 51, doi:10.1016/j.jad.2014.01.002 24581827

[r12] Cho MJ (2011). The Epidemiological Survey of Mental Disorders in Korea [in Korean]..

[r13] Dantzer R O’Connor JC Freund GG Johnson RW Kelley KW 2008 From inflammation to sickness and depression: when the immune system subjugates the brain. Nat Rev Neurosci 9 46 56, doi:10.1038/nrn2297 18073775 PMC2919277

[r14] Fonken LK Xu X Weil ZM Chen G Sun Q Rajagopalan S 2011 Air pollution impairs cognition, provokes depressive-like behaviors and alters hippocampal cytokine expression and morphology. Mol Psychiatry 16 987 995, doi:10.1038/mp.2011.76 21727897 PMC3270364

[r15] Gauderman WJ Urman R Avol E Berhane K McConnell R Rappaport E 2015 Association of improved air quality with lung development in children. N Engl J Med 372 905 913, doi:10.1056/NEJMoa1414123 25738666 PMC4430551

[r16] González HM Tarraf W Whitfield KE Vega WA 2010 The epidemiology of major depression and ethnicity in the United States. J Psychiatr Res 44 1043 1051, doi:10.1016/j.jpsychires.2010.03.017 20537350 PMC2963677

[r17] Juengling FD, Ebert D, Gut O, Engelbrecht MA, Rasenack J, Nitzsche EU (2000). Prefrontal cortical hypometabolism during low-dose interferon alpha treatment.. Psychopharmacology (Berl).

[r18] Jung CR Lin YT Hwang BF 2015 Ozone, particulate matter, and newly diagnosed Alzheimer’s disease: a population-based cohort study in Taiwan. J Alzheimers Dis 44 573 584, doi:10.3233/JAD-140855 25310992

[r19] Kim C Jung SH Kang DR Kim HC Moon KT Hur NW 2010 Ambient particulate matter as a risk factor for suicide. Am J Psychiatry 167 1100 1107, doi:10.1176/appi.ajp.2010.09050706 20634364

[r20] Kioumourtzoglou MA Austin E Koutrakis P Dominici F Schwartz J Zanobetti A 2015 PM_2.5_ and survival among older adults: effect modification by particulate composition. Epidemiology 26 321 327, doi:10.1097/EDE.0000000000000269 25738903 PMC4675621

[r21] Kioumourtzoglou MA Spiegelman D Szpiro AA Sheppard L Kaufman JD Yanosky JD 2014 Exposure measurement error in PM_2.5_ health effects studies: a pooled analysis of eight personal exposure validation studies. Environ Health 13 2, doi:10.1186/1476-069X-13-2 24410940 PMC3922798

[r22] Koton S Molshatzki N, Yuval, Myers V, Broday DM, Drory Y, et al. 2013 Cumulative exposure to particulate matter air pollution and long-term post-myocardial infarction outcomes. Prev Med 57 339 344, doi:10.1016/j.ypmed.2013.06.009 23777671

[r23] Lampron A Elali A Rivest S 2013 Innate immunity in the CNS: redefining the relationship between the CNS and Its environment. Neuron 78 214 232, doi:10.1016/j.neuron.2013.04.005 23622060

[r24] Lim YH Kim H Kim JH Bae S Park HY Hong YC 2012 Air pollution and symptoms of depression in elderly adults. Environ Health Perspect 120 1023 1028, doi:10.1289/ehp.1104100 22514209 PMC3404652

[r25] MacIntyre EA Karr CJ Koehoorn M Demers PA Tamburic L Lencar C 2011 Residential air pollution and otitis media during the first two years of life. Epidemiology 22 81 89, doi:10.1097/EDE.0b013e3181fdb60f 21030866

[r26] Meira-Machado L Cadarso-Suárez C Gude F Araújo A 2013 smoothHR: an R package for pointwise nonparametric estimation of hazard ratio curves of continuous predictors. Comput Math Methods Med 2013 745742, doi:10.1155/2013/745742 24454541 PMC3876718

[r27] MOE (Ministry of Environment) (2011). Annual Report of Ambient Air Quality in Korea, 2010 [in Korean]..

[r28] Moussavi S Chatterji S Verdes E Tandon A Patel V Ustun B 2007 Depression, chronic diseases, and decrements in health: results from the World Health Surveys. Lancet 370 851 858, doi:10.1016/S0140-6736(07)61415-9 17826170

[r29] Park JH, Lee JS, Lee JY, Hong JY, Kim SY, Kim SO (2006). Factors affecting national health insurance mass screening participation in the disabled [in Korean].. J Prev Med Public Health.

[r30] Park SK Adar SD O’Neill MS Auchincloss AH Szpiro A Bertoni AG 2015 Long-term exposure to air pollution and type 2 diabetes mellitus in a multiethnic cohort. Am J Epidemiol 181 327 336, doi:10.1093/aje/kwu280 25693777 PMC4339386

[r31] Persson R Björk J Ardö J Albin M Jakobsson K 2007 Trait anxiety and modeled exposure as determinants of self-reported annoyance to sound, air pollution and other environmental factors in the home. Int Arch Occup Environ Health 81 179 191, doi:10.1007/s00420-007-0204-1 17541626

[r32] Pope CA III Ezzati M Dockery DW 2009 Fine-particulate air pollution and life expectancy in the United States. N Engl J Med 360 376 386, doi:10.1056/NEJMsa0805646 19164188 PMC3382057

[r33] R Core Team (2015). R: A Language and Environment for Statistical Computing.. http://www.R-project.org.

[r34] Raison CL Miller AH 2011 Is depression an inflammatory disorder? Curr Psychiatry Rep 13 467 475, doi:10.1007/s11920-011-0232-0 21927805 PMC3285451

[r35] Rückerl R Hampel R Breitner S Cyrys J Kraus U Carter J 2014 Associations between ambient air pollution and blood markers of inflammation and coagulation/fibrinolysis in susceptible populations. Environ Int 70 32 49, doi:10.1016/j.envint.2014.05.013 24907704

[r36] Shelton RC, Miller AH (2011). Inflammation in depression: is adiposity a cause?. Dialogues Clin Neurosci.

[r37] Shin YJ, Yoon TH, Kim MH (2009). Health promotion strategies and programs development for health inequalities alleviation [in Korean]..

[r38] Szyszkowicz M Rowe BH Colman I 2009 Air pollution and daily emergency department visits for depression. Int J Occup Med Environ Health 22 355 362, doi:10.2478/v10001-009-0031-6 20197262

[r39] Szyszkowicz M Willey JB Grafstein E Rowe BH Colman I 2010 Air pollution and emergency department visits for suicide attempts in Vancouver, Canada. Environ Health Insights 4 79 86, doi:10.4137/EHI.S5662 21079694 PMC2978939

[r40] Tzivian L Winkler A Dlugaj M Schikowski T Vossoughi M Fuks K 2015 Effect of long-term outdoor air pollution and noise on cognitive and psychological functions in adults. Int J Hyg Environ Health 218 1 11, doi:10.1016/j.ijheh.2014.08.002 25242804

[r41] Valavanidis A Fiotakis K Vlachogianni T 2008 Airborne particulate matter and human health: toxicological assessment and importance of size and composition of particles for oxidative damage and carcinogenic mechanisms. J Environ Sci Health C Environ Carcinog Ecotoxicol Rev 26 339 362, doi:10.1080/10590500802494538 19034792

[r42] Wang Y Eliot MN Koutrakis P Gryparis A Schwartz JD Coull BA 2014 Ambient air pollution and depressive symptoms in older adults: results from the MOBILIZE Boston Study. Environ Health Perspect 122 553 558, doi:10.1289/ehp.1205909 24610154 PMC4050499

[r43] Weuve J Puett RC Schwartz J Yanosky JD Laden F Grodstein F 2012 Exposure to particulate air pollution and cognitive decline in older women. Arch Intern Med 172 219 227, doi:10.1001/archinternmed.2011.683 22332151 PMC3622279

[r44] Yoshida T, Osada Y, Kawaguchi T, Hoshiyama Y, Yoshida K, Yamamoto K (1997). Effects of road traffic noise on inhabitants of Tokyo.. J Sound Vib.

