# Supplementary material for: Long-Term Fine Particulate Matter Exposure and Major Depressive Disorder in a Community-Based Urban Cohort
Source: Environ Health Perspect. 2016 Apr 29;124(10):1547–53. doi: 10.1289/EHP192 (PMC5047772; doi:10.1289/EHP192)

Environ Health Perspect
DOI: 10.1289/EHP192

Note to readers with disabilities: *EHP* strives to ensure that all journal content is accessible to all readers. However, some figures and Supplemental Material published in *EHP* articles may not conform to 508 standards due to the complexity of the information being presented. If you need assistance accessing journal content, please contact ehp508@niehs.nih.gov. Our staff will work with you to assess and meet your accessibility needs within 3 working days.

Supplemental Material

Long-Term Fine Particulate Matter Exposure and Major Depressive Disorder in a Community-Based Urban Cohort

Kyoung-Nam Kim, Youn-Hee Lim, Hyun Joo Bae, Myounghee Kim, Kweon Jung, and Yun-Chul Hong

Table of Contents

Table S1. Descriptive characteristics of the study participants included and excluded in the analyses

Table S2. Distribution of the study participants (N = 27,270) and the annual $PM_{2.5}$ concentration ($\mu g/m^3$) in 2007 by districts in Seoul, Republic of Korea

Table S3. Hazard ratios of major depressive disorder for an increase of 10 $\mu g/m^3$ in the annual $PM_{2.5}$ concentration in 2007, stratified by sex and age

Table S4. Hazard ratios of major depressive disorder for an increase of 10 $\mu g/m^3$ in the annual $PM_{2.5}$ concentration in 2007, stratified by household income level

Table S5. Hazard ratios of major depressive disorder for an increase of 10 $\mu g/m^3$ in the moving average $PM_{2.5}$ concentration with a random effect of district

Table S6. Hazard ratios of depressive disorder for an increase of 10 $\mu g/m^3$ in the $PM_{2.5}$ concentration, stratified by different definitions of outcome

Table S7. Hazard ratios of major depressive disorder for an increase of 10 $\mu g/m^3$ in the $PM_{2.5}$ concentration after excluding or including participants who had major depressive disorder until December 31, 2007

Table S8. Hazard ratios of major depressive disorder for an increase of 10 $\mu g/m^3$ in the $PM_{2.5}$ concentration after excluding or including participants who died from intentional self-injury

Table S9. Hazard ratios of major depressive disorder for an increase of 10 μg/m^3 in the $PM_{2.5}$ concentration in the study population including those without health examination results

Figure S1. Flow chart of individuals who met the inclusion/exclusion criteria.

Figure S2. The annual average $PM_{2.5}$ concentrations between 2007 and 2010 in the 25 districts in Seoul.

Figure S3. Penalized regression spline model of the average $PM_{2.5}$ concentration in 2007 with a log-transformed hazard ratio. Solid lines, spline curve; shaded area, 95% confidence interval. The model is adjusted for sex, age (5-year group), household income, smoking status, alcohol consumption, regular exercise, the size of the population, proportion of the married person among population aged 15 years or older, economic and social environment satisfaction, the number of clients of the social welfare facilities per capita, and deprivation index in the district in which each participant resided.

Table S1. Descriptive characteristics of the study participants included and excluded in the analyses[a]

Variable	Included (n = 27,270)	Excluded (n = 58,488)	Total (n = 85,758)
Sex			
Male	14,782 (54)	28,602 (49)	43,384 (51)
Female	12,488 (46)	29,886 (51)	42,374 (49)
Age, years			
<15	0 (0)	11,259 (19)	11,259 (13)
15–29	2,213 (8)	16,959 (29)	19,172 (22)
30–39	4,288 (16)	7,802 (13)	12,090 (14)
40–49	8,059 (30)	9,489 (16)	17,548 (20)
50–59	7,445 (27)	6,299 (11)	13,744 (16)
60–69	4,050 (15)	3,591 (6)	7,641 (9)
70–79	1,215 (4)	1,949 (3)	3,164 (4)
>79	0 (0)	1,140 (2)	1,140 (1)
Household income, decile			
0–2	3,530 (13)	8,582 (15)	12,112 (14)
3–4	3,708 (14)	7,650 (13)	11,358 (13)
5–6	4,538 (17)	9,998 (17)	14,536 (17)
7–8	5,724 (21)	11,332 (19)	17,056 (20)
9–10	9,770 (36)	20,926 (36)	30,696 (36)
Size of the population in the district	445,095 (164,399)	445,095 (164,941)	445,095 (164,941)
Proportion of married persons among the population aged ≥15 years	0.56 (0.04)	0.56 (0.04)	0.56 (0.04)
Economic environment satisfaction[b]	4.85 (0.45)	4.85 (0.45)	4.85 (0.45)
Social environment satisfaction[b]	5.08 (0.52)	5.08 (0.52)	5.08 (0.52)
Number of clients of social welfare facilities[c]	8.85 (4.22)	8.85 (4.22)	8.85 (4.22)
Deprivation index	0.03 (2.53)	0.03 (2.53)	0.03 (2.53)

[a]Values are presented as n (%) or median (interquartile range). [b]Evaluated as a 10-point Likert scale. [c]Presented per 10,000 people. $PM_{2.5}$, particulate matter with an aerodynamic diameter ≤2.5 µm; SD, standard deviation.

Table S2. Distribution of the study participants (N = 27,270) and the annual $PM_{2.5}$ concentration ($\mu g/m^3$) in 2007 by districts in Seoul, Republic of Korea

Name of districts	Population in 2007, n (%)	Participants, n (%)	$PM_{2.5}$, mean
Dobong-gu	375,975 (3.7)	1,143 (4.2)	33.5
Dongdaemun-gu	376,421 (3.7)	1,001 (3.7)	20.3
Dongjak-gu	405,967 (4.0)	1,003 (3.7)	29.2
Eunpyeong-gu	459,196 (4.5)	1,339 (4.9)	28.3
Gangbuk-gu	345,478 (3.4)	918 (3.4)	28.7
Gangdong-gu	464,546 (4.6)	1,332 (4.9)	34.5
Gangnam-gu	560,958 (5.5)	1,306 (4.8)	30.9
Gangseo-gu	560,424 (5.5)	1,662 (6.1)	32.6
Geumcheon-gu	249,108 (2.4)	686 (2.5)	34.1
Guro-gu	420,542 (4.1)	1,205 (4.4)	32.8
Gwanak-gu	535,571 (5.3)	1,322 (4.9)	30.3
Gwangjin-gu	376,572 (3.7)	941 (3.5)	28.8
Jongno-gu	165,846 (1.6)	364 (1.3)	32.0
Jung-gu	130,044 (1.3)	295 (1.1)	28.7
Jungnang-gu	427,071 (4.2)	1,224 (4.5)	30.9
Mapo-gu	392,650 (3.9)	979 (3.6)	23.4
Nowon-gu	616,753 (6.1)	1,878 (6.9)	34.2
Seocho-gu	405,969 (4.0)	976 (3.6)	31.1
Seodaemun-gu	348,575 (3.4)	905 (3.3)	31.0
Seongbuk-gu	469,973 (4.6)	1,214 (4.5)	30.7
Seongdong-gu	333,535 (3.3)	838 (3.1)	26.4
Songpa-gu	623,876 (6.1)	1,756 (6.4)	29.5
Yangcheon-gu	503,650 (4.9)	1,450 (5.3)	26.4
Yeongdeungpo-gu	408,178 (4.0)	1,018 (3.7)	23.1
Yongsan-gu	235,832 (2.3)	515 (1.9)	28.8

$PM_{2.5}$, particulate matter with an aerodynamic diameter ≤ 2.5 μm; SD, standard deviation.

Table S3. Hazard ratios of major depressive disorder for an increase of 10 μg/m^3 in the annual $PM_{2.5}$ concentration in 2007, stratified by sex and age

	No.[a]	HR	95% CI	*P* for interaction
Sex[b]				
Male	369/14,782	1.52	1.07, 2.15	0.82
Female	604/12,488	1.40	1.08, 1.82	
Age, years[c]				
15–29	32/2,213	0.68	0.24, 1.95	0.63
30–39	62/4,288	1.34	0.59, 3.06	
40–49	247/8,059	1.65	1.07, 2.55	
50–59	298/7,445	1.47	1.00, 2.16	
60–69	222/4,050	1.23	0.80, 1.89	
70–79	112/1,215	1.81	0.98, 3.35	

[a]Number of events during the follow-up period/total number analyzed, stratified by sex and age. [b]The baseline hazard function was stratified by age, and the model was adjusted for household income, smoking status, alcohol consumption, regular exercise, size of the population, proportion of married persons among the population aged 15 years or older, economic and social environment satisfaction, number of clients of the social welfare facilities per capita, and deprivation index in the district in which each participant resided. [c]The baseline hazard function was stratified by sex, and the model was adjusted for household income, smoking status, alcohol consumption, regular exercise, size of the population, proportion of married persons among the population aged 15 years or older, economic and social environment satisfaction, number of clients of the social welfare facilities per capita, and deprivation index in the district in which each participant resided. $PM_{2.5}$, particulate matter with an aerodynamic diameter ≤2.5 μm; HR, hazard ratio; CI, confidence interval.

Table S4. Hazard ratios[a] of major depressive disorder for an increase of 10 μg/m^3 in the annual $PM_{2.5}$ concentration in 2007, stratified by household income level

Household income	No.[b]	HR	95% CI	*P* for interaction
0–3	193/5,297	2.14	1.29, 3.54	0.40
4–6	209/6,479	1.03	0.66, 1.62	
7–10	571/15,494	1.41	1.08, 1.85	

[a]Models were stratified by sex and age, and adjusted for household income, smoking status, alcohol consumption, regular exercise, size of the population, proportion of married persons among the population aged 15 years or older, economic and social environment satisfaction, number of clients of the social welfare facilities per capita, and deprivation index in the district in which each participant resided. [b]Number of events during the follow-up period/total number analyzed, stratified by underlying chronic diseases. $PM_{2.5}$, particulate matter with an aerodynamic diameter ≤2.5 μm; HR, hazard ratio; CI, confidence interval.

Table S5. Hazard ratios of major depressive disorder for an increase of 10 μg/m^3 in the moving average $PM_{2.5}$ concentration[a] with a random effect of district

Exposure	No.[b]	HR	95% CI
Model 1[c]	973/27,270	1.42	1.07, 1.87
Model 2[d]		1.42	1.07, 1.87
Model 3[e]		1.43	1.06, 1.93

[a]The 12-month moving average of the $PM_{2.5}$ concentration until an event or censor between 2007 and 2010 as time-varying exposure in time-dependent Cox models. [b]Number of events during the follow-up period/total number analyzed. [c]The models was stratified by sex and age, and unadjusted. [d]The models was stratified by sex and age, and adjusted for household income, smoking status, alcohol consumption, and regular exercise. [e]The models was stratified by sex and age, and adjusted for household income, smoking status, alcohol consumption, regular exercise, size of the population, proportion of married person among the population aged 15 years or older, economic and social environment satisfaction, number of clients of the social welfare facilities per capita, and deprivation index in the district in which each participant resided. $PM_{2.5}$, particulate matter with an aerodynamic diameter ≤2.5 μm; HR, hazard ratio; CI, confidence interval.

Table S6. Hazard ratios[a] of depressive disorder for an increase of 10 μg/m^3 in the $PM_{2.5}$ concentration, stratified by different definitions of outcome

	No.[b]	HR	95% CI
Depressive disorder I[c]	973/27,270		
$PM_{2.5}$ in 2007		1.44	1.17, 1.78
$PM_{2.5}$ between 2007 and 2010		1.59	1.02, 2.49
Moving average $PM_{2.5}$[d]		1.47	1.14, 1.90
Depressive disorder II[e]	1,147/26,722		
$PM_{2.5}$ in 2007		1.37	1.13, 1.66
$PM_{2.5}$ between 2007 and 2010		1.54	1.02, 2.31
Moving average $PM_{2.5}$[d]		1.42	1.12, 1.80
Depressive disorder III[f]	1,348/26,039		
$PM_{2.5}$ in 2007		1.29	1.08, 1.54
$PM_{2.5}$ between 2007 and 2010		1.59	1.09, 2.32
Moving average $PM_{2.5}$[d]		1.34	1.09, 1.67

[a]The models were stratified by sex and age, and adjusted for household income, smoking status, alcohol consumption, regular exercise, size of the population, proportion of married persons among the population aged 15 years or older, economic and social environment satisfaction, number of clients of the social welfare facilities per capita, and deprivation index in the district in which each participant resided. [b]Number of events during the follow-up period/total number analyzed. [c]Defined as the disease classification code F32.x with antidepressant prescription. [d]The 12-month moving average of the $PM_{2.5}$ concentration until an event or censor between 2007 and 2010 as time-varying exposure in time-dependent Cox models. [e]Defined as disease classification code F32.x. [f]Defined as disease classification codes F32.x, F33.x, F34.1, and F41.2. $PM_{2.5}$, particulate matter with an aerodynamic diameter ≤2.5 μm; HR, hazard ratio; CI, confidence interval.

Table S7. Hazard ratios[a] of major depressive disorder for an increase of 10 μg/m^3 in the $PM_{2.5}$ concentration after excluding or including participants who had major depressive disorder until December 31, 2007

	No.[b]	HR	95% CI
Participants who had MDD until December 31, 2007 were excluded	973/27,270		
$PM_{2.5}$ in 2007		1.44	1.17, 1.78
$PM_{2.5}$ between 2007 and 2010		1.59	1.02, 2.49
Moving average $PM_{2.5}$[c]		1.47	1.14, 1.90
Participants who had MDD until December 31, 2007 were included	1,481/28,863		
$PM_{2.5}$ in 2007		1.36	1.15, 1.61
$PM_{2.5}$ between 2007 and 2010		1.56	1.09, 2.24
Moving average $PM_{2.5}$[c]		1.43	1.18, 1.75

[a]The models were stratified by sex and age, and adjusted for household income, smoking status, alcohol consumption, regular exercise, size of the population, proportion of married persons among the population aged 15 years or older, economic and social environment satisfaction, number of clients of the social welfare facilities per capita, and deprivation index in the district in which each participant resided. [b]Number of events during the follow-up period/total number analyzed. [c]The 12-month moving average of the $PM_{2.5}$ concentration until an event or censor between 2007 and 2010 as time-varying exposure in time-dependent Cox models. $PM_{2.5}$, particulate matter with an aerodynamic diameter ≤2.5 μm; HR, hazard ratio; CI, confidence interval; MDD, major depressive disorder.

Table S8. Hazard ratios[a] of major depressive disorder for an increase of 10 μg/m^3 in the $PM_{2.5}$ concentration after excluding or including participants who died from intentional self-injury

	No.[b]	HR	95% CI
Participants who died from intentional self-injury were excluded	973/27,270		
$PM_{2.5}$ in 2007		1.44	1.17, 1.78
$PM_{2.5}$ between 2007–2010		1.59	1.02, 2.49
Moving average $PM_{2.5}$[c]		1.47	1.14, 1.90
Participants who died from intentional self-injury were included	991/27,270		
$PM_{2.5}$ in 2007		1.42	1.15, 1.75
$PM_{2.5}$ between 2007–2010		1.53	0.98, 2.38
Moving average $PM_{2.5}$[c]		1.45	1.12, 1.87

[a]The models were stratified by sex and age, and adjusted for household income, smoking status, alcohol consumption, regular exercise, size of the population, proportion of married persons among the population aged 15 years or older, economic and social environment satisfaction, number of clients of the social welfare facilities per capita, and deprivation index in the district in which each participant resided. [b]Number of events during the follow-up period/total number analyzed. [c]The 12-month moving average of the $PM_{2.5}$ concentration until an event or censor between 2007 and 2010 as time-varying exposure in time-dependent Cox models. $PM_{2.5}$, particulate matter with an aerodynamic diameter ≤2.5 μm; HR, hazard ratio; CI, confidence interval.

Table S9. Hazard ratios of major depressive disorder for an increase of 10 μg/m^3 in the $PM_{2.5}$ concentration in the study population including those without health examination results

	No.[a]	HR	95% CI
Model 1[b]	2,003/69,999		
$PM_{2.5}$ in 2007		1.43	1.25, 1.63
$PM_{2.5}$ between 2007–2010		1.52	1.14, 2.05
Moving average $PM_{2.5}$[c]		1.20	1.01, 1.42
Model 2[d]	2,003/69,999		
$PM_{2.5}$ in 2007		1.34	1.16, 1.55
$PM_{2.5}$ between 2007–2010		1.41	1.03, 1.93
Moving average $PM_{2.5}$[c]		1.19	1.00, 1.41

[a]Number of events during the follow-up period/total number analyzed. [b]Stratified by sex and age, and unadjusted. [c]The 12-month moving average of the $PM_{2.5}$ concentration until an event or censor between 2007 and 2010 as time-varying exposure in time-dependent Cox models. [d]Stratified by sex and age, and adjusted for household income, smoking status, alcohol consumption, regular exercise, size of the population, proportion of married persons among the population aged 15 years or older, economic and social environment satisfaction, number of clients of the social welfare facilities per capita, and deprivation index in the district in which each participant resided. $PM_{2.5}$, particulate matter with an aerodynamic diameter ≤2.5 μm; HR, hazard ratio; CI, confidence interval.

Figure S1. Flow chart of individuals who met the inclusion/exclusion criteria.

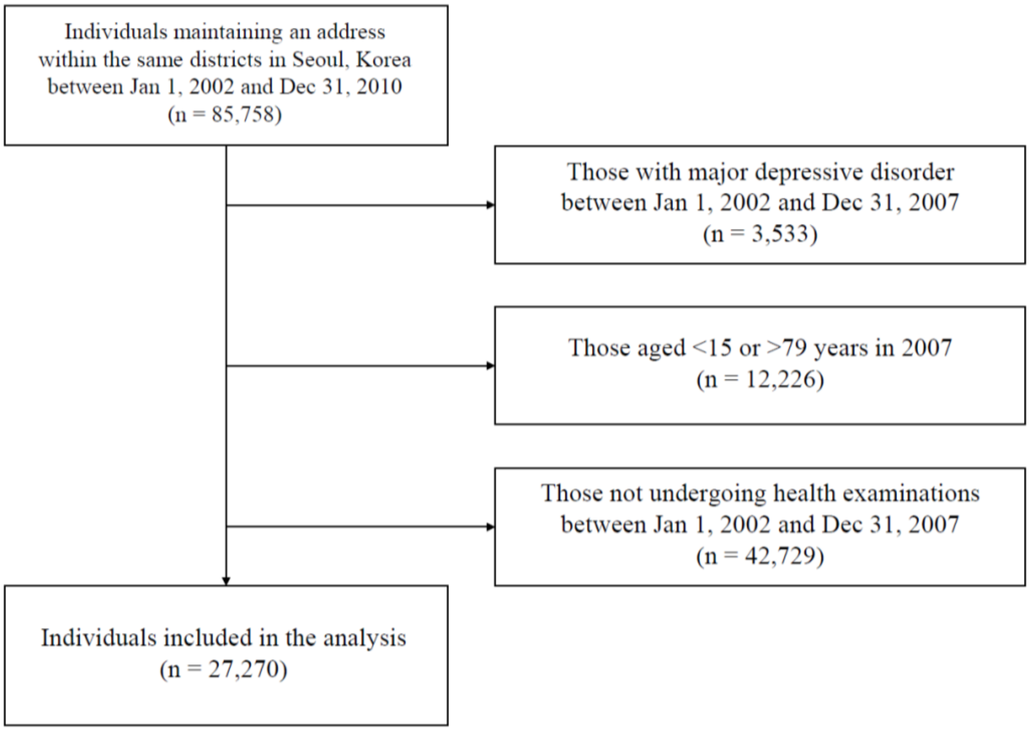

Figure S2. The annual average $PM_{2.5}$ concentrations between 2007 and 2010 in the 25 districts in Seoul.

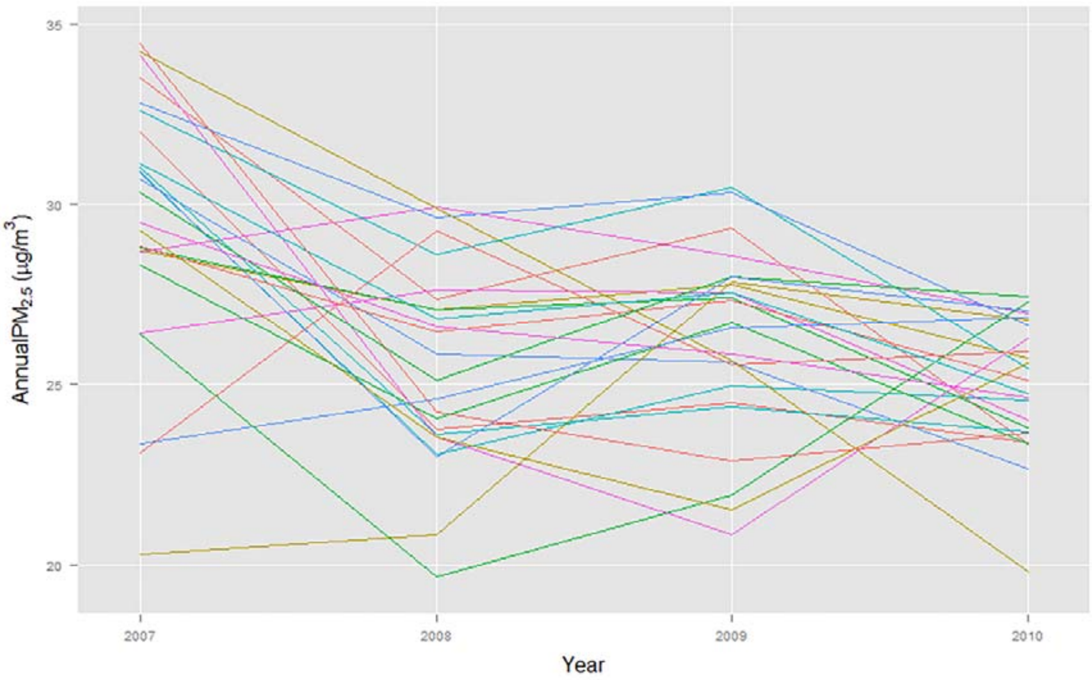

Figure S3. Penalized regression spline model of the average $PM_{2.5}$ concentration in 2007 with a log-transformed hazard ratio. Solid lines, spline curve; shaded area, 95% confidence interval. The model is adjusted for sex, age (5-year group), household income, smoking status, alcohol consumption, regular exercise, the size of the population, proportion of the married person among population aged 15 years or older, economic and social environment satisfaction, the number of clients of the social welfare facilities per capita, and deprivation index in the district in which each participant resided.

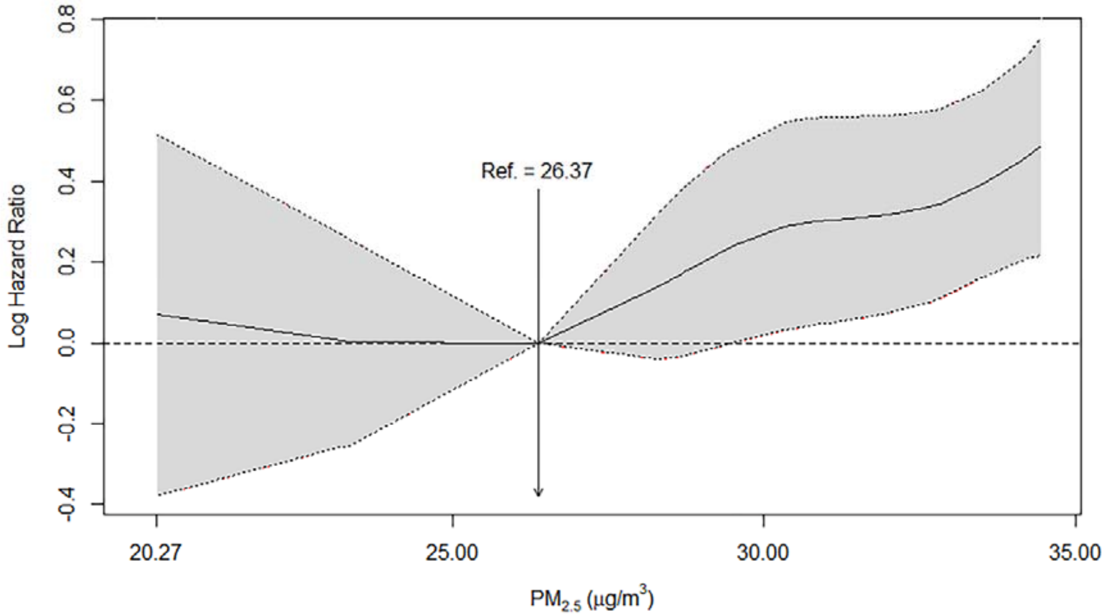

Supplement: (442 KB) PDF [file EHP192.s001.acco.pdf]
